# The association between iron deficiency and muscle mass/strength in patients undergoing maintenance hemodialysis

**DOI:** 10.3389/fnut.2025.1628038

**Published:** 2025-10-02

**Authors:** Yan Liu, Min Chen, Jieyi Zhang, Jie Luo, Tingyu Chen, Xiaoyan Zeng, Huibin Nie

**Affiliations:** ^1^Department of Clinical Nutrition, Chengdu First People’s Hospital, Chengdu, Sichuan, China; ^2^Department of Nephrology, Chengdu First People’s Hospital, Chengdu, Sichuan, China

**Keywords:** iron deficiency, muscle strength, muscle mass, maintenance hemodialysis, sarcopenia, transferrin saturation, bioelectrical impedance analysis

## Abstract

**Background:**

Patients with end-stage renal disease (ESRD) are at high risk of losing muscle mass and strength, especially those undergoing maintenance hemodialysis (MHD). Iron deficiency (ID) may exacerbate this condition, however, its relationship with muscle mass/strength remains largely unclear. Therefore, we aim to investigate the association between ID status and muscle mass/strength in Chinese patients undergoing hemodialysis.

**Methods:**

This cross-sectional study was conducted at a hemodialysis center in southwest China from September to December 2022. Iron status was assessed by plasma ferritin and transferrin saturation (TSAT). Muscle strength was measured by handgrip strength (HGS), and muscle mass was evaluated using the appendicular skeletal muscle index (ASMI). Linear regression and subgroup analysis with interaction terms were performed to explore the associations.

**Results:**

269 participants on MHD aged 18–85 years were included, 21.9% (59/269) had ID. Participants with ID exhibited significantly lower handgrip strength, but similar ASMI, compared to those without ID (grip strength: 21.6 ± 8.7 vs. 24.3 ± 8.9, ASMI: 6.5 ± 1.2 vs. 6.5 ± 1.0). After adjusting for potential covariates, ID was negatively associated with handgrip strength (*β* = −2.21, 95% CI: −4.08 to −0.34, *p* = 0.021). Subgroup analysis confirmed the stability of this result. Interestingly, when stratified by overweight status, ID was significantly associated with ASMI in overweight participants (*β* = 0.50, 95% CI: 0.17–0.84, *p* = 0.004), while no association was observed in non-overweight participants (*β* = −0.20, 95% CI: −0.44 to 0.05, *p* = 0.240; *p*-value for interaction = 0.004).

**Conclusion:**

These findings demonstrate that ID is significantly associated with reduced muscle strength in patients on MHD in southwest China. Additionally, ID was significantly associated with muscle mass only in overweight participants. These results provide strong support for the importance of individualized iron—repletion strategies to preserve muscle health in patients on MHD, particularly those who are overweight, although further verification in prospective studies is needed.

## 1 Introduction

Patients with end-stage renal disease (ESRD) undergoing renal replacement therapy were at high risk of sarcopenia (1), characterized by the loss of muscle mass and strength. This condition was particularly prevalent in patients undergoing hemodialysis, with an incidence of approximately 31% (2), underscoring its clinical significance. Reduced muscle mass and strength were strongly associated with increased risks of cardiovascular events (1), hospitalizations, and all-cause mortality in patients undergoing hemodialysis (3). In contrast, greater muscle mass and strength were linked to improved survival outcomes (4), highlighting the importance of muscle preservation in enhancing prognosis.

Multiple factors contributed to sarcopenia in patients with ESRD, including the accumulation of uremic toxins, acidic metabolic byproducts, malnutrition, amino acid losses during dialysis, and chronic inflammation (5). Emerging evidence also suggested a potential association between iron deficiency and reduced muscle mass or strength in populations with chronic disease (6), including the elderly (7). However, whether iron deficiency directly affected muscle mass or strength in patients on MHD remained unclear.

Iron was essential for maintaining skeletal muscle energy metabolism and overall function (6). It played a critical role in oxygen uptake, transport, storage, and oxidative metabolism in both cardiac and skeletal muscle cells (8). In cardiomyocytes, iron deficiency impaired oxidative phosphorylation and myocardial contractility, leading to reduced skeletal muscle strength and increased lactate production in patients with heart failure (8, 9). Iron supplementation had been shown to improve aerobic glycolysis in these patients (10). Additionally, iron deficiency was strongly associated with reduced muscle mass in the general population (7), further emphasizing its widespread impact on muscle health. Although iron deficiency was common among patients on MHD due to minor blood loss during dialysis (11, 12), whether it contributed to muscle wasting through altered iron metabolism in patients undergoing hemodialysis remained uncertain.

In this study, we aimed to evaluate the association between iron deficiency and muscle mass and strength among patients on MHD. Furthermore, we sought to explore whether this association was modified by key nutritional and clinical factors, including age, overweight status, anemia status, iron supplementation, and erythropoiesis-stimulating therapies.

## 2 Materials and methods

### 2.1 Study design and participants

**Figure 1:**
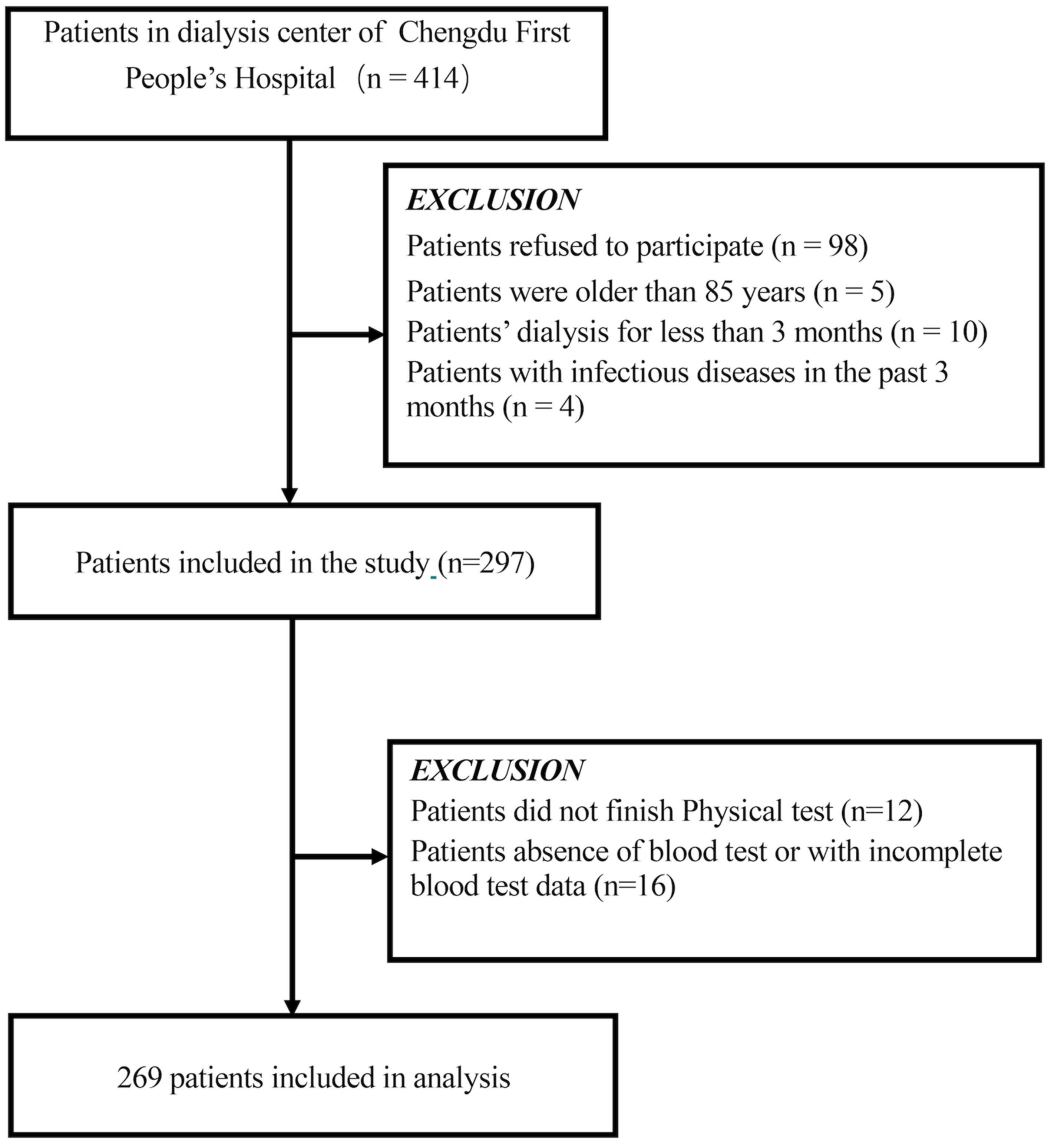
Schematic representation of the participant selection process and distribution of participant groups. MHD, maintenance hemodialysis.

This cross-sectional study was conducted at Chengdu First People’s Hospital from September to December 2022. Patients aged 18–85 years who had undergone maintenance hemodialysis for at least 3 months were included. Exclusion criteria were as follows: refusal to participate (*n* = 98), dialysis duration less than 3 months (*n* = 10), history of infectious disease within the past 3 months (*n* = 4), inability to complete the physical test due to cerebrovascular or severe cardiopulmonary disease (*n* = 12), and absence or incompleteness of blood test data (*n* = 16). The flowchart of participant enrollment is shown in Figure 1. Ultimately, 269 participants were included in the analysis. All patients underwent hemodialysis three times per week (4 h per session) using bicarbonate dialysate and cellulose acetate or polysulfone dialysis membranes. The majority of patients (95%) were receiving antihypertensive medications and standard ESRD treatments, including phosphate binders, erythropoiesis-stimulating therapies, iron and vitamin D supplements.

### 2.2 Ethics statement

All participants provided informed consent. The study was approved by the Ethics Committee of Chengdu First People’s Hospital (approval number: 2022KT003).

### 2.3 Measurements

#### 2.3.1 Definition of iron deficiency

Venous blood samples were collected prior to a midweek pre-dialysis session. Serum ferritin levels were measured using electrochemiluminescence immunoassay (ECLIA). Serum iron and unsaturated iron-binding capacity (UIBC) were assessed via colorimetric assay. Transferrin saturation (TSAT) was calculated as: 100 × serum iron (μmol/L) / serum iron (μmol/L) + UIBC (μmol/L). Iron deficiency was defined as serum ferritin <200 μg/L and TSAT <20% in patients on MHD, as previously suggested (13–16).

#### 2.3.2 Measurement of muscle strength and muscle mass

Muscle strength was assessed using handgrip strength (HGS), as previously described (17). HGS was measured prior to the midweek hemodialysis session using an electronic grip strength meter (EH101, Xiangshan Inc., Guangdong, China). Patients were instructed to hold the device vertically with the elbow positioned by the side of the body and to exert maximum voluntary force. The analysis was based on the highest value obtained from three consecutive measurements on the non-fistula side or dominant arm.

Muscle mass was assessed using the appendicular skeletal muscle index (ASMI), as previously reported (17). Appendicular skeletal muscle mass (ASM) was measured using a multi-frequency bioelectrical impedance analysis device (InBody S10, BioSpace, Seoul, Korea), which operates at six frequencies (1, 5, 50, 250, 500, and 1,000 kHz) and distinguishes between intracellular and extracellular fluid compartments. To ensure measurement accuracy and minimize fluid-related artifacts inherent to individuals undergoing hemodialysis, all assessments were performed in the standing position at least 30 min following the completion of a midweek dialysis session, when patients had achieved their estimated dry weight. Electrode placement followed the manufacturer’s 8-point protocol: electrodes were placed on the thumbs and middle fingers of both hands, as well as on the flat areas below the ankles bilaterally. Participants were instructed to avoid food intake and strenuous physical activity for at least 4 h prior to measurement. The device’s built-in algorithm adjusts for hydration status by calculating the extracellular water-to-total body water (ECW/TBW) ratio, enhancing reliability in this patient population. ASMI was calculated as ASM (kg)/height^2^ (m^2^).

#### 2.3.3 Covariates

Patient demographics, including age, sex, and dialysis duration, were obtained from medical records. Comorbid conditions such as chronic obstructive pulmonary disease, hypertension, coronary heart disease, diabetes mellitus, arthritis, stroke, and tumors were self-reported by participants and verified against medical records. The age-adjusted Charlson Comorbidity Index (CCI) was calculated using the method described by Charlson et al. (18).

Laboratory data were collected before a midweek pre-dialysis session and included neutrophil and lymphocyte counts, hemoglobin, plasma lipids, albumin, alkaline phosphatase, creatinine, uric acid, urea nitrogen, serum calcium, serum phosphorus, intact parathyroid hormone (iPTH), high-sensitivity C-reactive protein (hs-CRP), and β2-microglobulin. Albumin-corrected calcium levels were calculated using the formula: serum calcium (mmol/L) + [40 − albumin (g/L)] × 0.02. The single-pool urea clearance index (spKt/V) was calculated using pre- and post-dialysis urea nitrogen values, following the formula described by Daugirdas (19).

The nutritional status of the patients was assessed using the Malnutrition Inflammation Score (MIS), as previously described (20). This evaluation included four main components: medical history (covering changes in dry weight, dietary habits, gastrointestinal symptoms, functional status, and comorbidities), physical examination, body mass index (BMI), and laboratory tests. The total MIS ranged from 0 (normal nutritional status) to 30 (severe malnutrition), based on the sum of 10 component scores.

BMI was calculated as dry weight (kg) divided by height squared (m^2^). Overweight status was defined according to the World Health Organization classification for the Asian population (21). Participants were categorized into two groups based on BMI: non-overweight (BMI < 23.0 kg/m^2^) and overweight (BMI ≥ 23.0 kg/m^2^).

### 2.4 Statistical analysis

Participant characteristics were described as mean (standard deviation [SD]) for continuous variables with a normal distribution or median (interquartile range [IQR]) for skewed distributions. Parametric data were compared using the independent samples t-test, while non-parametric data were analyzed using the Mann–Whitney U test. Categorical variables were expressed as proportions and compared using the chi-square test or Fisher’s exact test, as appropriate.

Multivariable linear regression analyses were performed to examine the associations between iron deficiency and muscle mass or muscle strength. Missing data for covariates were less than 3%; specifically, four participants (1.49%) had missing plasma lipid values, five (1.86%) had missing TSAT, and one each (0.37%) had missing hs-CRP or iPTH. Participants with missing data were excluded from the analyses. Variables identified in previous studies (22, 23) or considered clinically relevant were included in the multivariable models. Covariates were also retained in the final model if they were potential confounders that changed the estimated association between iron deficiency and muscle mass or strength by more than 10%. Three models were constructed: Model 1 was unadjusted; Model 2 was adjusted for covariates based on theoretical relevance and previous findings (22, 23); and Model 3 was further adjusted for covariables that changed the matched OR by at least 10% when it was added to this model.

To assess whether the relationship between iron deficiency and muscle mass or strength was consistent across subgroups, interaction and subgroup analyses were conducted according to age group, overweight status, anemia status, iron supplementation, and erythropoiesis-stimulating therapy strategies. Interactions were tested using the likelihood ratio test. Additionally, due to the skewed distribution of ferritin levels, a log_2_ transformation was applied to assess its association with muscle mass.

All statistical analyses were performed using R statistical software (The R Foundation) and Free Statistics software version 1.9. All tests were two-tailed, and a *p*-value <0.05 was considered statistically significant.

## 3 Results

### 3.1 Characteristics of the study population

From September to December 2022, a total of 269 patients aged 18–85 years were enrolled in this study. The mean age of participants was 59.8 ± 13.9 years, and 53.2% were male. Among all participants, 21.9% (59/269) were classified as having iron deficiency. Clinical characteristics and laboratory data of the study population are summarized in Table 1. There were no significant differences in sex, age, age-adjusted CCI, Malnutrition Inflammation Score (MIS), or the prevalence of diabetes mellitus between patients without ID and those with ID (all *p* > 0.05). However, participants with ID had significantly lower handgrip strength (21.6 ± 8.7 kg vs. 24.3 ± 8.9 kg, *p* = 0.038) and fat mass (12.3 [8.9, 16.4] kg vs. 15.4 [9.2, 20.4] kg, *p* = 0.019) compared to those without ID. In terms of laboratory findings, patients with ID exhibited significantly lower levels of triglycerides (1.6 [1.2, 2.4] mmol/L vs. 2.0 [1.3, 3.0] mmol/L, *p* = 0.045), ferritin (45.6 [20.9, 93.8] μg/L vs. 161.7 [72.1, 339.0] μg/L, *p* < 0.001), and transferrin saturation (TSAT) (16.9 [13.3, 18.7]% vs. 28.5 [23.8, 35.2]%, *p* < 0.001), while high-density lipoprotein cholesterol (HDL-c) was marginally higher compared to patients without ID (1.0 ± 0.4 mmol/L vs. 0.9 ± 0.3 mmol/L, *p* = 0.002).

**Table 1:** Characteristics of the study participants.

Variables	Total (*n* = 269)	Non-ID (*n* = 210)	ID (*n* = 59)	*p* value
Sex, *n* (%)				0.935
Male	143 (53.2)	112 (53.3)	31 (52.5)	
Female	126 (46.8)	98 (46.7)	28 (47.5)	
Age (years)	59.8 ± 13.9	59.7 ± 13.8	60.0 ± 14.5	0.885
Duration (month)	56.2 ± 45.0	58.2 ± 44.8	48.9 ± 45.4	0.162
Age-adjusted CCI	4.8 ± 1.6	4.9 ± 1.6	4.8 ± 1.6	0.666
DM, *n* (%)				0.693
No	193 (72.6)	149 (72)	44 (74.6)	
Yes	73 (27.4)	58 (28)	15 (25.4)	
MIS	4.8 ± 3.0	4.8 ± 3.1	4.8 ± 2.8	0.867
Height (cm)	160.4 ± 8.6	160.5 ± 8.7	160.1 ± 8.5	0.720
Weight (kg)	57.5 ± 11.7	58.0 ± 11.6	55.6 ± 12.2	0.162
BMI (kg/m^2^)	22.3 ± 3.7	22.5 ± 3.8	21.6 ± 3.6	0.096
Fat mass (kg)	14.6 (9.0, 19.8)	15.4 (9.2, 20.4)	12.3 (8.9, 16.4)	0.019
ASMI (kg/m^2^)	6.5 ± 1.0	6.5 ± 1.0	6.5 ± 1.2	0.853
Grip strength (kg)	23.7 ± 8.9	24.3 ± 8.9	21.6 ± 8.7	0.038
Gait speed (m/s)	0.8 ± 0.2	0.8 ± 0.2	0.8 ± 0.3	0.267
Hemoglobin (g/L)	108.2 ± 17.5	108.9 ± 16.8	105.8 ± 19.6	0.223
HDL-c (mmol/L)	0.9 ± 0.3	0.9 ± 0.3	1.0 ± 0.4	0.002
LDL-c (mmol/L)	2.3 ± 0.7	2.3 ± 0.8	2.2 ± 0.6	0.235
Total cholesterol (mmol/L)	3.7 ± 0.9	3.7 ± 1.0	3.6 ± 0.8	0.375
Triglyceride (mmol/L)	1.9 (1.3, 2.8)	2.0 (1.3, 3.0)	1.6 (1.2, 2.4)	0.045
Albumin (g/L)	39.3 ± 3.5	39.5 ± 3.4	38.6 ± 3.7	0.102
BUN (mg/dL)	21.2 ± 7.3	21.4 ± 7.3	20.5 ± 7.4	0.402
Creatinine (μmol/L)	871.4 ± 262.7	886.0 ± 263.9	820.1 ± 253.9	0.089
Uric acid (μmol/L)	404.8 ± 113.1	410.6 ± 110.6	384.8 ± 120.5	0.123
P (mmol/L)	1.6 ± 0.5	1.6 ± 0.5	1.7 ± 0.6	0.378
Ca (mmol/L)^a^	2.6 ± 0.5	2.6 ± 0.5	2.7 ± 0.5	0.140
iPTH (pg/mL)	246.2 (150.5, 425.2)	246.2 (159.6, 399.9)	242.8 (109.0, 508.6)	0.921
AKP (U/L)	91.0 (75.0, 113.8)	90.0 (75.0, 110.0)	99.0 (73.5, 124.0)	0.289
β2 microglobulin (ug/L)	42.8 (34.5, 50.5)	42.8 (34.5, 50.3)	44.0 (34.9, 51.0)	0.892
spKt/V	1.4 (1.2, 1.6)	1.4 (1.2, 1.6)	1.4 (1.2, 1.7)	0.573
hs-CRP (mg/dL)	3.2 (1.4, 6.7)	3.0 (1.4, 6.5)	3.3 (1.7, 7.6)	0.408
Serum ferritin (μg/L)	108.9 (52.6, 288.3)	161.7 (72.1, 339.0)	45.6 (20.9, 93.8)	<0.001
TSAT (%)	26.1 (19.4, 33.7)	28.5 (23.8, 35.2)	16.9 (13.3, 18.7)	<0.001

CCI, Charlson Comorbidity Index; DM, diabetes mellitus; MIS, malnutrition inflammation score; BMI, body mass index; ASMI, appendicular skeletal muscle mass index; HDL-c, high-density lipoprotein cholesterol; LDL-c, low-density lipoprotein cholesterol; BUN, blood urea nitrogen; iPTH, intact parathyroid hormone; AKP, alkaline phosphatase; spKt/V, single-pool urea clearance index; hs-CRP, high-sensitivity C-reactive protein; TSAT, transferrin saturation.

a We modified serum Ca with serum albumin.

### 3.2 Association between iron deficiency and muscle strength

Univariate analyses showed significant positive correlations between hemoglobin, albumin, and TSAT levels and handgrip strength (all *p* < 0.001) (Table 2). Multivariable linear regression models were used to further evaluate the association between ID status and handgrip strength. In the unadjusted model, ID was significantly associated with lower handgrip strength (*β* = −2.72, 95% CI: −5.28 to −0.16, *p* = 0.038). After adjusting for age, sex, albumin, hemoglobin, BMI, and HDL-c, the association remained statistically significant (*β* = −2.21, 95% CI: −4.08 to −0.34, *p* = 0.021) (Table 3). Subgroup analyses according to age, overweight status, anemia status, iron supplementation, and erythropoiesis-stimulating therapies consistently supported a negative association between ID and handgrip strength (Supplementary Figure 1).

**Table 2:** Univariate analysis of associations between hemoglobin, iron indices, nutritional and inflammatory markers, and muscle strength/mass.

Variables	Grip strength		ASMI	
	Coefficient (95% CI)	*p*	Coefficient (95% CI)	*p*
Hemoglobin (g/L)	0.1 (0.04, 0.16)	<0.001	0 (0, 0.01)	0.475
HDL-c (mmol/L)	−4.72 (−8.4, −1.03)	0.012	−0.72 (−1.15, −0.29)	0.001
LDL-c (mmol/L)	−0.15 (−1.6, 1.31)	0.842	−0.11 (−0.28, 0.06)	0.197
Triglyceride (mmol/L)	0.04 (−0.55, 0.63)	0.893	0.01 (−0.06, 0.08)	0.835
Total cholesterol (mmol/L)	−0.52 (−1.68, 0.63)	0.371	−0.15 (−0.28, −0.01)	0.030
Albumin (g/L)	0.96 (0.68, 1.25)	<0.001	0.07 (0.04, 0.11)	<0.001
NLR	−0.01 (−0.56, 0.55)	0.983	0.02 (−0.05, 0.08)	0.566
hs-CRP (mg/dL)	−0.13 (−0.24, −0.02)	0.023	0 (−0.01, 0.01)	0.903
β2 microglobulin (ug/L)	−0.04 (−0.12, 0.05)	0.420	−0.01 (−0.02, 0)	0.022
Log_2_ transformed ferritin (μg/L)	−0.04 (−0.72, 0.63)	0.896	−0.06 (−0.14, 0.02)	0.152
TSAT (%)	0.22 (0.13, 0.31)	<0.001	0.01 (0, 0.02)	0.090

HDL-c, high-density lipoprotein cholesterol; LDL-c, low-density lipoprotein cholesterol; ASMI, appendicular skeletal muscle mass index; NLR, neutrophil-to-lymphocyte ratio; hs-CRP, high-sensitivity C-reactive protein; TSAT, transferrin saturation; 95% CI, 95% confidence interval.

**Table 3:** Multivariable analysis of associations between iron deficiency and muscle strength/mass.

Variables	Grip strength			ASMI		
	Standardized coefficient (*β*)	95% CI	*p* value	Standardized coefficient (*β*)	95% CI	*p* value
Model 1	−2.72	−5.28 to −0.16	0.038	−0.03	−0.33 to 0.27	0.853
Model 2	−2.25	−4.05 to −0.44	0.015	0.01	−0.22 to 0.23	0.952
Model 3	−2.21	−4.08 to −0.34	0.021	0.07	−0.10 to 0.24	0.421

Model 1: Crude model, adjust for nothing.

Model 2: Adjusted for age, sex and albumin.

Model 3: Adjusted for age, sex and albumin and variables when added to this model, changed the matched odds ratio by at least 10%, including hemoglobin, BMI, and HDL-c.

95% CI, 95% confidence interval; BMI, body mass index; ASMI, appendicular skeletal muscle mass index; HDL-c, high-density lipoprotein cholesterol.

### 3.3 Association between iron deficiency and muscle mass

Univariate analysis demonstrated a significant positive correlation between albumin levels and ASMI (*p* < 0.001) (Table 2). However, multivariable regression analysis did not reveal a significant association between iron deficiency and ASMI in the overall study population. Adjusting for age, sex, albumin, and confounders identified by a ≥10% change in matched odds ratios yielded nonsignificant results (*β* = 0.07, 95% CI: −0.10 to 0.24, *p* = 0.421) (Table 3). Subgroup analyses based on age, anemia status, iron supplementation, and erythropoiesis-stimulating therapies consistently supported the absence of a significant association (Supplementary Figure 2). Notably, a significant interaction was observed between overweight status and iron deficiency in relation to muscle mass (*p*-value for interaction = 0.004).

Among overweight participants, ID was positively associated with ASMI (*β* = 0.50, 95% CI: 0.17–0.84, *p* = 0.004), whereas no significant association was observed in non-overweight individuals (*β* = −0.20, 95% CI: −0.44 to 0.05, *p* = 0.240) (Table 4). Additionally, the association between log_2_-transformed ferritin levels and ASMI was evaluated in patients on MHD stratified by overweight status. In overweight individuals, higher log_2_-transformed ferritin levels were significantly associated with lower ASMI (*β* = −0.13, 95% CI: −0.22 to −0.05, *p* = 0.003), while no significant association was observed in non-overweight participants (*β* = 0.03, 95% CI: −0.04 to 0.10, *p* = 0.380) (Table 5 and Supplementary Figure 3). These findings underscore the complex interplay between iron-related parameters, muscle mass, and overweight status in patients on MHD.

**Table 4:** Interaction between iron deficiency and ASMI by overweight status in patients on MHD.

Variables	Non-overweight (*n* = 158)		Overweight (*n* = 111)		*p* for interaction
	Standardized coefficient (*β*) (95% CI)	*p*-value	Standardized coefficient (*β*) (95% CI)	*p*-value	
Non-ID	0 (ref)		0 (ref)		0.004
ID	−0.20 (−0.44 to 0.05)	0.240	0.50 (0.17–0.84)	0.004	

Analysis adjusted for age, sex and albumin and variables when added to this model, changed the matched odds ratio by at least 10%, including hemoglobin and HDL-c.

95% CI, 95% confidence interval; ASMI, appendicular skeletal muscle mass index; ID, iron deficiency; MHD, maintenance hemodialysis; HDL-c, high-density lipoprotein cholesterol.

**Table 5:** Interaction between ferritin and ASMI by overweight status in patients on MHD.

Variables	Non-overweight (*n* = 158)		Overweight (*n* = 111)		*p* for interaction
	Standardized coefficient (*β*) (95% CI)	*p*-value	Standardized coefficient (*β*) (95% CI)	*p*-value	
Log_2_ transformed ferritin	0.03 (−0.04 to 0.10)	0.380	−0.13 (−0.22 to −0.05)	0.003	0.002

Analysis adjusted for age, sex, albumin, hemoglobin, and HDL-c.

95% CI, 95% confidence interval; ASMI, appendicular skeletal muscle mass index; MHD, maintenance hemodialysis; HDL-c, high-density lipoprotein cholesterol.

## 4 Discussion

In this cross-sectional study involving participants aged 18–85 years undergoing MHD, we investigated the relationship between iron deficiency and both muscle strength and muscle mass. Our findings reveal a robust association between iron deficiency and reduced muscle strength, a correlation that remains significant even after accounting for hemoglobin levels and other potential confounders. Subgroup analyses based on age, overweight status, anemia status, iron supplementation, and erythropoiesis-stimulating therapy strategies showed no statistically significant interactions. However, a notable interaction was observed between overweight status and iron deficiency in relation to muscle mass. Specifically, iron deficiency was positively associated with ASMI in overweight participants, whereas no such association was found in the non-overweight group.

Skeletal muscle strength impairment, including decreased performance and diminished exercise capacity, is commonly observed in patients with chronic diseases (24), with a primary focus on energetics and a consequent loss of muscle oxidative capacity (25). Experimental data from *in vitro* and animal studies suggest that the effects of iron deficiency on skeletal muscle include a disrupted selection of energy substrates and altered catabolic pathways (26, 27). Iron deficiency shifts the balance from oxidative phosphorylation to anaerobic fermentation by altering the morphology of the inner mitochondrial membrane cristae (6), affecting key mitochondrial oxidative phosphorylation enzymes, and inducing uncoupling of the mitochondrial electron transport chain (28). Additionally, iron deficiency leads to lipid droplet formation in skeletal muscle cells, which promotes lipid peroxidation and cellular damage (29).

Recent clinical studies have increasingly highlighted the relationship between iron deficiency and muscle strength. Bekfani et al. emphasized a significant association between iron deficiency and impaired skeletal muscle functional capacity in patients with heart failure (9). Observational and longitudinal cohort studies in patients with acute stroke have demonstrated a notable correlation between iron deficiency and decreased muscle strength (30). Furthermore, iron deficiency has been linked to poor functional outcomes following rehabilitation (31). A cohort study in patients with chronic obstructive pulmonary disease (COPD) found that individuals with iron deficiency reported more frequent exacerbations and exhibited a trend toward poorer exercise tolerance (32). Our study extends and supports these earlier findings in individuals aged 18–85 years with End-Stage Renal Disease (ESRD) undergoing MHD in Southeast China. We observed that iron deficiency was independently associated with reduced muscle strength, even after adjusting for potential confounders such as age, sex, albumin, hemoglobin, BMI, and HDL-c levels. Furthermore, this association was consistently observed across various subgroups based on age, overweight status, anemia status, iron supplementation, and erythropoiesis-stimulating therapy strategies. Further research is needed to validate these findings and to explore the complex relationship and underlying mechanisms.

On the other hand, emerging studies have provided evidence linking iron deficiency with muscle mass, although the results have been inconsistent. A recent population-based cohort study involving 8,592 adults suggested a significant association between iron deficiency and reduced muscle mass, independent of hemoglobin levels and other potential confounders (7). However, our study did not find a statistically significant association between iron deficiency and muscle mass in patients undergoing hemodialysis in Southeast China. This finding differs from previous reports. Nonetheless, subgroup analysis revealed an interaction between iron deficiency, overweight status, and muscle mass. Specifically, in overweight participants on MHD, we observed that iron deficiency was positively associated with ASMI, and log_2_ transformed serum ferritin was negatively associated with ASMI. These associations were not observed in the non-overweight group. Consistent with our findings, a cross-sectional study using the NHANES database demonstrated that serum ferritin was negatively linked with muscle mass after adjusting for potential confounders (22). Additionally, a study involving 300 patients undergoing hemodialysis showed a significant negative correlation between log_2_ transformed ferritin and muscle quality (23).

The observation that iron deficiency exhibited a positive correlation with ASMI exclusively in overweight participants suggests a complex interaction between adiposity and iron metabolism in the regulation of muscle mass. Several mechanisms may contribute to this phenomenon. First, iron is stored in muscle as myoglobin, and obese individuals may exhibit greater iron accumulation in muscle tissues, as shown by imaging and transcriptomic data across multiple cohorts (33). Second, chronic low-grade inflammation and oxidative stress associated with obesity may alter muscle iron handling, leading to mitochondrial dysfunction and increased proteolytic activity via the ubiquitin-proteasome pathway (34). Third, insulin resistance, commonly observed in obese individuals, has been linked to both iron overload and impaired muscle anabolism (35, 36). Animal studies have shown that iron-deficient diets can mitigate high-fat-diet-induced insulin resistance and preserve muscle function (36). Therefore, the paradoxical association observed in overweight patients may reflect a compensatory or protective effect of iron deficiency in the setting of iron-related metabolic stress. These hypotheses warrant further mechanistic investigation in populations on MHD, where disturbances in iron metabolism and body composition frequently coexist.

This study provides valuable insights into the association between iron deficiency and muscle strength and mass in patients on MHD. However, several limitations must be acknowledged. Firstly, the cross-sectional design of the study limits the ability to draw causal inferences. Therefore, future research will employ interventional trials modifying iron supplementation strategies—particularly in subgroups such as overweight versus non-overweight patients, to evaluate whether correcting iron deficiency can mitigate muscle deterioration. This approach will provide stronger evidence on the relationship between iron deficiency and muscle deterioration in this vulnerable population. Additionally, the study population was confined to a single center in Southwest China, genetic, dietary, and clinical practice differences may exist across different ethnic and geographical populations. Therefore, the generalizability of our findings to other populations, such as those in Western countries, may be limited. Multi-center, international studies are needed to validate our results in more diverse cohorts. Secondly, it is important to recognize that iron metabolism and inflammatory pathways play critical roles in muscle metabolism. To minimize the impact of acute inflammatory states, individuals with acute infections were excluded from the study. In future cohort studies, we intend to include a broader range of iron regulatory proteins and inflammatory mediators to better elucidate the mechanisms linking iron metabolism, inflammation, and muscle dysfunction. Furthermore, we did not assess physical activity levels in our study. Given that reduced physical activity is a well-recognized contributor to sarcopenia and has been independently associated with both muscle mass and strength (37), this omission may have introduced residual confounding. Future studies should incorporate standardized assessments of physical activity to better delineate its influence on the relationship between iron deficiency and muscle outcomes in populations on MHD. Another limitation is exclusive use of BMI to define overweight status without incorporating measures of central adiposity, such as waist circumference. Although current KDOQI Clinical Practice Guideline for Nutrition in Chronic Kidney Disease: 2020 Update (38) recommend the use of BMI-based weight categories in patients with CKD, BMI has limitations—it cannot differentiate between lean and fat mass or assess visceral adiposity. Future studies should incorporate more refined body composition metrics to better characterize overweight-related risks in populations on MHD.

Despite these limitations, the study has several strengths. Notably, subgroup analyses were conducted to examine potential confounders affecting muscle mass and strength, including age, anemia, overweight status, iron supplementation and erythropoiesis-stimulating therapy strategies. Previous literature (39) suggests that intravenous iron therapy improves exercise capacity more effectively than oral iron in patients with anemic, iron-deficient heart failure. However, our subgroup analysis revealed that iron supplementation strategies and anemia status did not significantly influence the association between iron deficiency and muscle mass or strength. This distinction underscores the complexity of iron’s role in muscle physiology and highlights the need for further investigation into the differential impacts of oral versus intravenous iron supplementation on muscle parameters in patients on MHD. Overall, the rigorous analytical approach and consideration of relevant confounders enhance the robustness of our findings, contributing meaningful knowledge to the field of CKD-related muscle dysfunction. In addition, our findings underscore the need for individualized iron supplementation strategies in patients on MHD. While iron supplementation may be beneficial for improving muscle strength in patients undergoing hemodialysis with iron deficiency, caution is warranted in those who are overweight. We found that iron deficiency was positively associated with muscle mass in overweight patients. Given the pro-inflammatory state of adiposity (34), aggressive iron therapy in this subgroup might potentially exacerbate oxidative stress without conferring a benefit to muscle mass. Clinicians could therefore integrate body composition and inflammatory status into iron therapy decision-making, particularly for overweight patients. Further studies are needed to determine the optimal iron supplementation strategy for patients on MHD, taking into account their body composition and other clinical factors.

## 5 Conclusion

This study demonstrated a robust association between iron deficiency and reduced muscle strength, with a noteworthy interaction observed between overweight status and iron deficiency concerning muscle mass. Specifically, iron deficiency was positively associated with ASMI in overweight participants. These findings suggest that iron supplementation should be approached with caution in overweight patients undergoing hemodialysis. Further cohort studies are warranted to investigate the potential role of iron deficiency in both muscle mass and muscle function in patients on MHD.

## Data Availability

The original contributions presented in the study are included in the article/Supplementary material, further inquiries can be directed to the corresponding author.
