# Supplementary material for: The association between iron deficiency and muscle mass/strength in patients undergoing maintenance hemodialysis
Source: Front Nutr. 2025 Oct 2;12:1628038. doi: 10.3389/fnut.2025.1628038 (PMC12529934; doi:10.3389/fnut.2025.1628038)
Supplement: Supplementary file 1 [file Supplementary_file_1.docx]

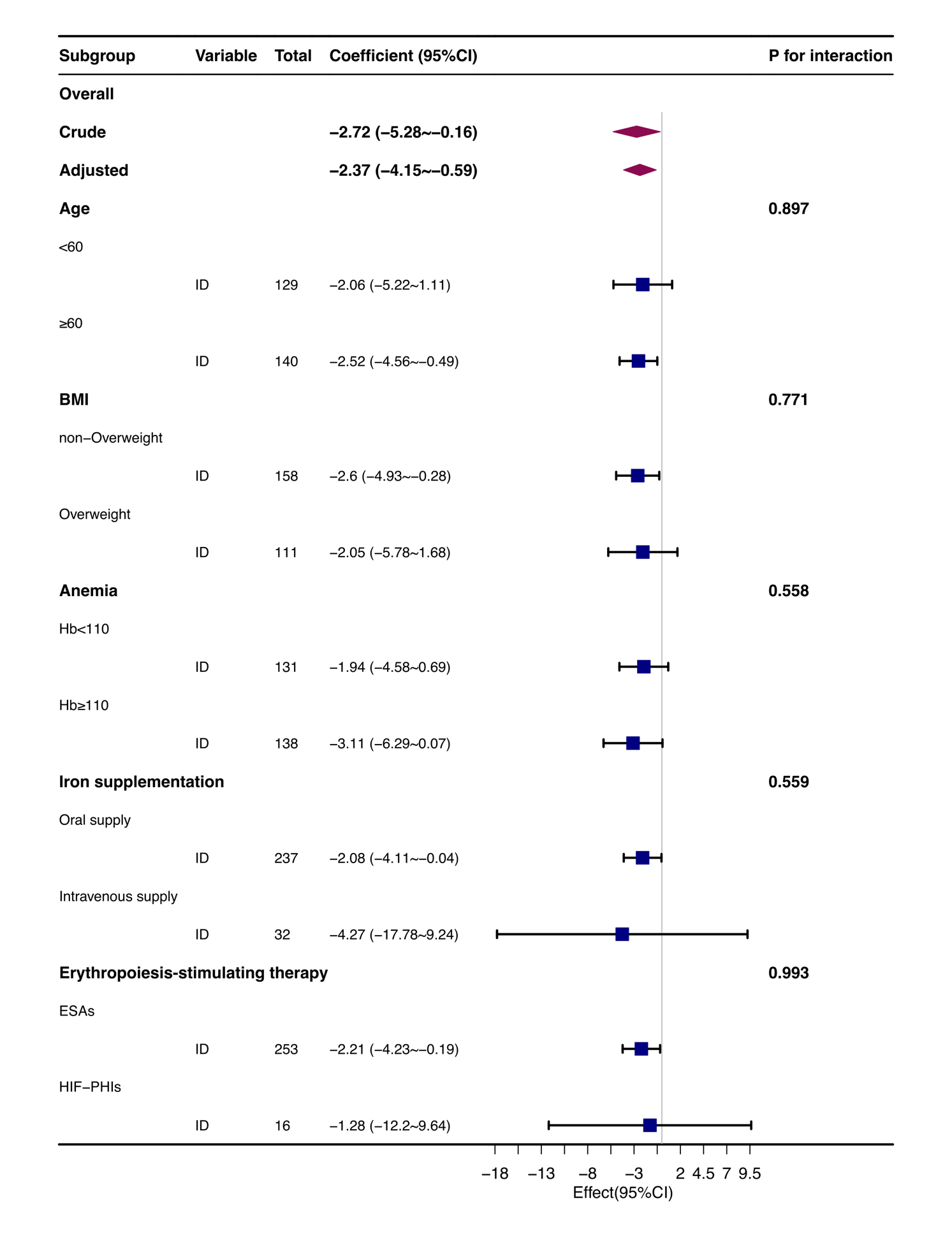


**Supplementary Figure 1.** Subgroup Analysis of the Associations between Iron Deficiency and Grip Strength in Patients on MHD.

Note: The forest plot presents regression coefficients (squares) with 95% confidence intervals (horizontal lines) from multivariable linear models adjusted for sex, albumin, and HDL-c, unless otherwise indicated. Diamonds represent overall estimates for crude and adjusted models.

Subgroup analyses were conducted according to: age (<60 vs. ≥60 years), BMI category (non-overweight [<23 kg/m²] vs. overweight [≥23 kg/m²]), anemia status (hemoglobin <110 g/L vs. ≥110 g/L), iron supplementation route (oral vs. intravenous), and erythropoiesis-stimulating therapy type (ESAs vs. HIF-PHIs). The p value for interaction represents the likelihood of interaction between the variable and the handgrip.

The negative association between iron deficiency and grip strength was consistent across all the subgroups, with no statistically significant interactions (all *p*-value for interaction >0.05).

Abbreviations: ID, iron deficiency; MHD, maintenance hemodialysis; BMI, body mass index; ESAs: Erythropoiesis-Stimulating Agents; HIF-PHIs: Hypoxia-Inducible Factor Prolyl Hydroxylase Inhibitors; HDL-c, high-density lipoprotein cholesterol; CI, confidence interval.


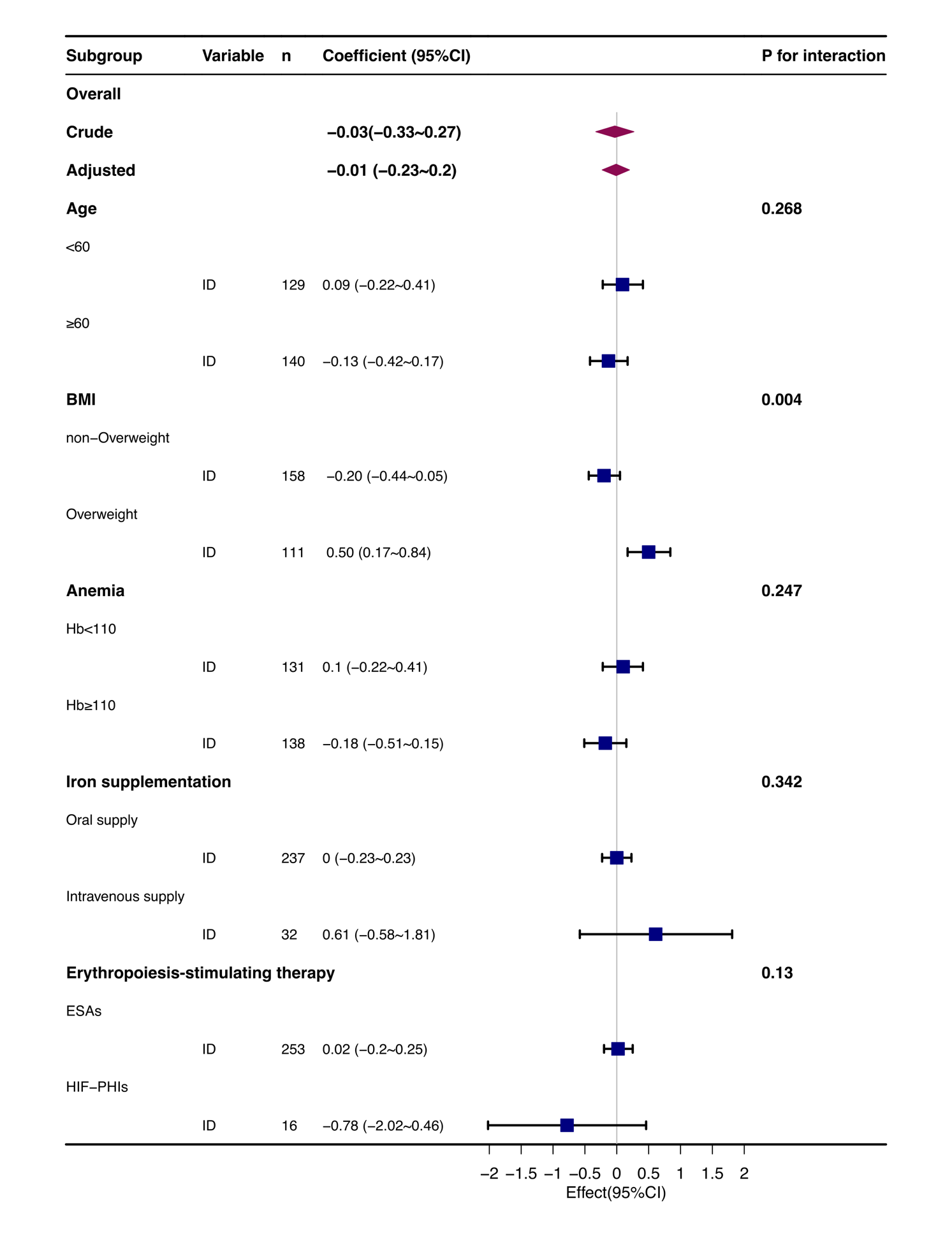


**Supplementary Figure** **2.** Subgroup Analysis of the Associations between Iron Deficiency and ASMI in Patients on MHD.

Note: The forest plot presents regression coefficients (squares) with 95% confidence intervals (horizontal lines) from multivariable linear models adjusted for sex, albumin, and HDL-c, unless otherwise indicated. Diamonds represent overall estimates for crude and adjusted models.

Subgroup analyses were conducted according to: age (<60 vs. ≥60 years), BMI category (non-overweight [<23 kg/m²] vs. overweight [≥23 kg/m²]), anemia status (hemoglobin <110 g/L vs. ≥110 g/L), iron supplementation route (oral vs. intravenous), and erythropoiesis-stimulating therapy type (ESAs vs. HIF-PHIs). The p value for interaction represents the likelihood of interaction between the variable and the ASMI.

A significant interaction was observed for BMI category (*p for interaction* = 0.004). No other subgroup showed a statistically significant interaction.

Abbreviations: ID, iron deficiency; MHD, maintenance hemodialysis; BMI, body mass index; ASMI, appendicular skeletal muscle mass index; ESAs: Erythropoiesis-Stimulating Agents; HIF-PHIs: Hypoxia-Inducible Factor Prolyl Hydroxylase Inhibitors; HDL-c, high-density lipoprotein cholesterol; CI, confidence interval.

**
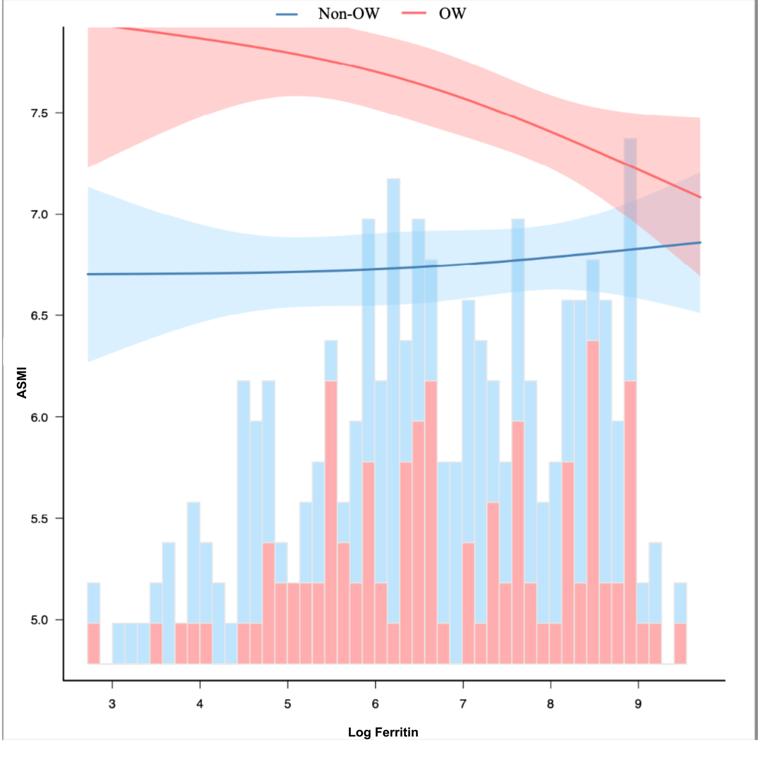
**

**Supplementary Figure 3.** Interaction between Log_2_-transformed Ferritin and ASMI by Overweight Status in Patients on MHD.

Note: Ferritin values were log_2_-transformed to normalize the distribution. The analysis was adjusted for age, sex, albumin, hemoglobin, and HDL-c.

In the overweight group, higher ferritin concentrations were associated with a downward trend in ASMI, whereas no obvious trend was observed in the non-overweight group. This pattern is consistent with the significant interaction between BMI category and iron status observed in subgroup analyses (see Supplementary Figure 2).

Abbreviations: BMI, body mass index; OW, overweight; ASMI, appendicular skeletal muscle mass index; HDL-c, high-density lipoprotein cholesterol.
